# Can Artificial Intelligences Suffer from Mental Illness? A Philosophical Matter to Consider

**DOI:** 10.1007/s11948-016-9783-0

**Published:** 2016-06-28

**Authors:** Hutan Ashrafian

**Affiliations:** Imperial College London, 10th Floor QEQM-Building, Praed Street, London, W2 1NY UK

**Keywords:** Artificial intelligence, Robot, Mental, Illness, Psychiatry, Psychology, Philosophy, Psychoanalysis, Psychopathology, Consciousness, Sentience, Rationality

## Abstract

The potential for artificial intelligences and robotics in achieving the capacity of consciousness, sentience and rationality offers the prospect that these agents have minds. If so, then there may be a potential for these minds to become dysfunctional, or for artificial intelligences and robots to suffer from mental illness. The existence of artificially intelligent psychopathology can be interpreted through the philosophical perspectives of mental illness. This offers new insights into what it means to have either robot or human mental disorders, but may also offer a platform on which to examine the mechanisms of biological or artificially intelligent psychiatric disease. The possibility of mental illnesses occurring in artificially intelligent individuals necessitates the consideration that at some level, they may have achieved a mental capability of consciousness, sentience and rationality such that they can subsequently become dysfunctional. The deeper philosophical understanding of these conditions in mankind and artificial intelligences might therefore offer reciprocal insights into mental health and mechanisms that may lead to the prevention of mental dysfunction.

## Hypothetical Case

The post-battle collection of military robots had identified that 5 of the autonomous-firing AI robots had logged incapacities for continued battle. Diagnostics of their physical attributes revealed no faults, however the robots expressed inability to participate in combat. Robot A complained of ‘lack of energy’ despite normal battery power. In addition there was a description of self-criticism, sadness and a consideration for auto-execution. Robot B displayed recurrent avoidance of any combat-like situation and had become to demonstrate obsessive–compulsive symptoms, particularly in controlling his immediate work-environment variables such as temperature and humidity despite being built as an all-environment combatant. This robot had also asked for spiritual salvation, asking to discuss his worries with a priest. Robot C had become unable to work in a team and was describing an altered sense of self, there was even the description of perceiving commands or voices when none had been issued.

Robot system analytics were unable to identify any underlying dysfunction although a chance investigation through a human diagnostic system identified the diagnoses of Depression for Robot A, Anxiety with Post Traumatic Stress for Robot B and Psychosis for Robot C.

## Introduction

The nature of artificial intelligence has led to the discussion of whether robots have agency. The subsequent questions of whether artificial intelligences (AIs) will demonstrate consciousness, sentience and sapience have all remained controversial. This continues to vex researchers in this field as the definitions for these terms are not yet universally or formally defined. Alan Turing was able to bypass these semantics by focusing on the practicality of how an artificially intelligent unit could perform against a human according to his eponymous Turing Test (Ashrafian et al. 2015). As the practical reality of introducing ever more advanced artificially intelligent technologies approaches, the reality of whether they demonstrate ‘true’ consciousness or whether they only mimic consciousness may become a conceptual issue alongside the burgeoning reality of AI’s demonstrating ever more human-like mental abilities (Ashrafian 2015a, b). There is however a concomitant issue of whether AIs will demonstrate human-like mental disability.

In practical terms sentience has been considered as the capacity to experience pain and pleasure as a proxy for self-awareness, which in turn has led to anticipation for a definition that has yet to materialize and this view continues to be challenged. To-date the definition of consciousness remains vague and the seemingly objective property of ‘intelligence’ remains to be hotly contested by mankind. Consequently Turing’s circumvention of these “semantics” through the supposedly objective Turing Test suggests that saying that if something passes the Turing test there is nothing more that we need to know about intelligence. Asking the question of what intelligence is according to Turing therefore seems pointless and supports a very Wittgensteinian view. Understanding the very nature of intelligence however has now manifested as a germane issue to clarify both philosophical and practical differentiators between human and artificial intelligence. The Turing Test and its modern interpretations remain controversial and still polarize and divide the scientific community. Through this maelstrom of disagreed and inadequate definitions, the fields of psychiatry and clinical psychology have arguably made some solid advances in at least identifying and classifying mental illness, although pinpointing causes has proven more challenging.

## Philosophy of Mental Illness Applied to AI

In the hypothetical case presented above, the robots could be considered to suffer from mental diseases when considered through a human diagnostic lens, however (i) can they also be considered as robot mental illnesses (or are they simply a human overlay from an underlying mimicking effect)? (ii) would these robots suffer from these diseases in the same way as humans? (iii) considering that the AIs would not have been designed, built or programmed to suffer from any cognitive dysfunction, would such a finding give insight into the philosophy of mental illness?

In the case described, the robots did not demonstrate any material changes in their physical structure or processing analysis so that when considered by skeptical Szaszian theory (Szasz 1978; Pickard 2009), they cannot formally be considered to suffer from mental illness. This is because there was no material or structural evidence for their mental dysfunction so that they cannot be suffering from an underlying disease or pathology, rather this is seemingly their choice of behaviour. According to this school, no behavior or misbehavior can be considered a ‘disease’ (‘a malfunction of the human body’ or a state of ‘not being at ease’) that Thomas Szasz believed would be derived from clear objective physical, physiological or biochemical tests. Here mental diseases exist as a metaphor societal dysfunction. According to Szasz and Foucault, one of the primary drivers for the adoption of these conceptions of mental diseases was used as a use for societal force and control.

If we did assume that these robots were suffering from mental illness, then arguments against Szaszian theory would include the notion that either we were unable to test for the material changes in the robots to adequately diagnose any underlying mental illness, or that the robots did have minds and that material analysis may not be a measure of any underling mental disorders.

The possibility of robotic mental health problems refloats the older theories of substance dualism in mental illness where these disorders were categorized as the ‘non-physical substances’ of the mind. Here Cartesian dualism suggests that these diseases exist in the non-physical elements of ‘soul’, ‘mind’ and ‘consciousness’, which in themselves are not clearly defined concepts. The concept of dualism suggests that the non-physical substance in the brain can interact with the physical substance. In the case of mental illness, disorders in non-physical components associated with the brain would be translated in the physical segments. One particular argument for dualism is that of the philosophical zombie (or p-zombie) mind experiment of David Chalmers (Chalmers 1996). Here the philosophical existence of a p-zombie (where a fully functioning body can exist without a conscious mind) renders the possibility of consciousness as a natural phenomenon. The concept of a p-zombie has been considered analogous to a human-like robot with human physical abilities but without its mental faculties, however this does not consider the possibility of consciousness in an advanced artificial intelligence. A p-zombie then by definition could not suffer from mental illness, whereas a robot who had the ability of consciousness and mental health may also be liable to mental illness.

There are also additional issues, for example, mentally ill patients are sentient, conscious with measurable intelligence. The problem is whether the presence of these three traits in an entity means that mental illness can emerge. Whilst it remains controversial distinguishing philosophical zombies in all cases from humans, I argue that an entity with consciousness must then be able to exhibit mental disorder, which may at some level therefore exist as a disorder when consciousness is present.

The concept of eliminative materialism championed by Churchland (Churchland 1988) explained that mental disorders do not exist as disorders of mental states, which are non-existent, but rather they derive from underlying neurobiological processes (in the case of artificial intelligences, these would however be neuroelectronic processes). This theory contradicts the infallibility thesis forwarded by Descartes and supported by Searle where mental states and therefore disorders are infallibly real. Eliminative materialism negates the infallibility thesis through the fact that infallibility theory’s representation in folk psychological models of the world are falsifiable, rendering the infallibility thesis also falsifiable. Arguments against eliminative materialism include those of self-refutation where the actual thought of a concept of eliminativism may in itself be a ‘mental state’ of belief that does not exist. The notion of qualia existing as individual and subjective instances of conscious experience also dispute eliminativism negating of common-sense mental states. Reductive materialist oppose eliminative materialists by suggesting each metal state does have an organic neurobiological origin, whereas revisionary materialists propose that emerging understanding of neurobiology will offer concomitant appreciation of any underlying neurobiological origins of mental disorders.

Traditionally Cartesian thought focuses on the uniquely human ability of language, which is now reappraised in view of the burgeoning language skills of modern artificial intelligences. There are however several arguments against substance dualism; (i) biological knowledge including patients with brain splitting surgery (at the corpus callosum) which has identified biological locations for different components of what had traditionally been considered parts of the mind. (ii) the subjectivity of mental disorders (iii) the role of naturalism explaining all mental states and (iv) it does not account for mental causation in performing tasks.

If therefore, robots were to develop symptoms of mental illness, this would identify three factors: (1) could these robots have been inadvertently programmed to have mental disorders; if so could these be easily reversed by corrective programming? (2) if the robots had consciousness and free will, did they develop mental illness de-novo (against their original programming). (3) By extension, if artificial intelligences had independently (against their original programming) developed mental illness, could this represent their initial transition to human-like consciousness and then subsequently to mental disease?

A more recent approach to the philosophy of metal illness includes that of George Graham who argues that mental illness occurs when the psychological or mental capacities necessary for flourishing health fail to allow the subsistence of our wellbeing. He suggests that these capacities are mental due to their characteristics of philosophical intentionality that contributes to consciousness. They include effectual elements such as the ability to act in the world, to form goals, shape behaviors to fit them, to make choices and to have emotional commitment. According to Graham, mental illnesses arise when these capacities are “gummed up” and limit mental functionality and subsequently wellbeing. A core paradigm of this theory is built on the work of several twentieth century philosophers linking the attributes of philosophical intentionality with rationality and consciousness. It then expands by identifying the mechanisms of mental illness deriving from the breakdown of rational mental relationships from mechanical processes and that ultimately all mental illnesses are likely to demonstrate a varying degree of both mechanical and philosophical intentional (mental) constituents. For example, in psychosis, there are some elements of rationality (in terms of capacity for reasoning) although clearly there is a mechanical disorder of mental functioning due to rationality being ‘gummed up’.

Such a breakdown of mental illness between (i) philosophical intentionality mental rationality and (ii) mechanical processes in the brain render this theory a modern take on mind–body dualism where it endeavors to answer both questions of whether minds are material (a metaphysical juxtaposition) and whether they act in mental disease (addressing mental causation). He therefore adopts a functionalism or non-reductive physicalist approach suggesting an independent existence of the mind, based upon, but separate from, inherent neurobiological (material) processes. If artificial intelligences develop symptoms of human-like mental illness, this could lend support to a functionalist approach. Here despite totally different underlying neurobiological processes, if robot minds and human minds were to develop similar mental disorders under similar conditions it might offer some new insights. The very nature of humans coding artificial intelligences may render them liable to similar mental states and disorders of those states in particular circumstances. Of course, this might not be the case, as by definition and build, humans and robots are different; why should machines’ exhibiting similar symptoms under similar circumstances (given their different material structures) lend solid insight into human mental disorder? The issues of similarity in mental disorders might lead to misconceptions regarding similar circumstances and symptoms and how can these be determined without falling into a circular fallacy that disorders imply similar minds which imply similar disorders. Nonetheless The act of using human-designed language and human thought processes to design any technology, however objectively may render any of our creations fundamentally biased to our human view of the universe. The fact we code with a human language suggests a Wittgensteinian derivation of human-like attributes inherent in any humanly designed object (even through subconscious means). This is because human computational semantics is not fundamentally computational and can lack relevance to formal computational methods (Wilks 2008, 2011). Human generated language can lack perspicuity, so that even when the designers are purposefully actualizing their thoughts away from humanity, such as the building of combatant robots, human-type thought processes pervade any underlying coding.

One method to achieve a reductionist and purely neurobiological view of mental disorders can derive from an evolutionary perspective. Accordingly mental disorders can be considered as by-products or ‘spandrels’ of evolution or even as a direct result of evolution. For example, cerebral structure has been reported as being evolved to accommodate language in hominid advancement and the neurobiological mechanism offering this ability may also account for for psychosis (Crow 2008; Brune 2004). Alternatively, depression is associated with several genes that govern inflammation, so that the behavioral social withdrawal noted in this disorder has been explained as a mechanism to minimize infectious diseases (Raison et al. 2006) within a population.

Clearly robots were not evolved in the same Darwinian fashion as humans over a long period of natural selection (even though some engineers may have used some ‘evolutionary principles’ at some point in their pathway of machine genesis), rather they generated de-novo computers with various levels of processing power to offer the potential of cognition. As a result, the finding of mental illness in artificial intelligences may represent either their inherent human-like mental actions which may directly or indirectly represent their human designers. It may also represent a mimicking of humanity or result from a de-novo genesis of disease in their rational and conscious minds.

One approach to characterize mental function derives from a Dual Process Theory (DPT) methodology, in which mental reasoning processes have been proposed to derive from two distinct function types. This comprises System 1 and System 2 parts of brain functioning; the former offering autonomous (automatic, fast, evolutionarily old, ‘unconscious’) versus the latter considered (reasoned, thought out, slow, evolutionarily recent, ‘conscious’) aspects of brain activities. This approach has been increasingly applied to mental illness, so that for example higher autism traits (Brosnan et al. 2016) and cognitive vulnerability to depression (Haeffel et al. 2007) are characterised by a consistent bias towards deliberative reasoning of System 2. Although Dual Process Theory does have limitations, particularly those assuming that all mental functions can be mapped on to (i) two principal cognitive systems, and (ii) that these 2 systems interact with each other to represent mental function; it does nevertheless offer a practical approach to assess the complex system of cognition in each conscious individual. Additionally such an approach may help contribute to coping and treatment strategies in minimizing mental dysfunction in humans (Kato 2015) and potentially even artificial intelligences.

In the event that we were able to show that there were robots who demonstrated consciousness and subsequently suffered from symptoms of mental illness, then it would be incumbent on mankind to perform the humane action of treating them. For such a finding would necessitate the core values of human society’s humane duty to another rational and conscious being; from a practical and importantly from a moral and ethical stance. By creating artificial intelligences with these abilities, we are also duty bound to offer them a duty of care along with their inherent rights with which we are increasingly considering (Ashrafian 2015b, c).

The next step in such a consideration would include by what means we would offer treatment for these mentally ill robots. Would similar approaches to human mental disease work? or would there have to be machine equivalents? For example, psychiatric disease interventions in humans incudes, actively not treating an illness, pharmacotherapy, psychotherapy, counseling, and even surgery. In the case of robots this may include changing hardware or re-coding software. This would have ethical implications as for artificial intelligences, there may be the potential to totally recode a robot mind and delete or modify a robots programming to remove its symptoms of mental illness; however, would there be an additional risk of irrevocably changing the underlying conscious and rational individual. Reprogramming a conscious mind to alter it irreversibly in such a way with or possibly even without consent could be considered as an infringement of inherent rights and associated with both moral and ethical crimes.

One situation could arise where robots experiencing “mental disease” would automatically shut down (‘spanner in the works’); this may arise from such severe malfunction that prevents the robot from continuing its activities, or alternatively it may have been pre-programmed pre-emptively to shut down in the event of a malfunction. In practice, just as human mental diseases do not automatically result in mortality or complete cessation of activity, the automatic shutting down of a ‘conscious’ robot with mental dysfunction may not take place.

It can be envisaged that AIs and robots can be programed to handle uncertainty and ambiguity based on algorithms that accept risk and the problems associated with a choice that is deleterious. This of course may not be adequate for the diverse complexity of interactions between sentient individuals in preventing mental dysfunction. Just as in humans, the effect of mental illness in AIs or robots may cause direct pain, although it is uncertain how would such a being would react, particularly if they were not designed to accommodate the ‘sense of pain’.

The case for Robot B also introduces added complexity, for this robot also asked for relief of symptoms by asking for a priest. This request for spiritual salvation introduces the conceptual association between mind and religion. The topic of human religion in AI has previously been addressed (Bainbridge 2006), however what type to priest would Robot B request? What religion or belief system? And what form of religious therapy for management of its mental symptoms. Whilst the concept of ‘divine commands’ (Bringsjord and Taylor 2012) are used in AI programming, the case does offer concepts of AI-specific religions, priests and religious therapies.

If these robots are afforded the rights of medical or psychiatric treatment for their disease, an additional question is who should deliver that therapy? For example a simplistic delineation would be whether a fellow AI or a human should carry out this therapy. This however detracts from the more fundamental medical aim of identifying the most appropriate individual or group to offer that therapy; whatever their biological or engineered origin. By doing so, the act of treating an artificial intelligence for mental illness could be perceived that we are finally recognizing AI’s to have consciousness. It can be argued that managing a robot or AI’s physical dysfunctions as a purely mechanical or engineering task, but the deployment of mental health therapy for artificial intelligences necessities the prerequisite that these individuals have minds that are capable of dysfunction. The fact that they are considered to have minds that can potentially demonstrate psychopathology may well therefore be an unexpected but nonetheless a robust downstream indicator of whether a mind is conscious or not.

In essence therefore, I suggest that mental illness is a ‘consequential derivative’ or emergent property in a subset of conscious individuals. In humans this may arise from either acquired or inherited elements and may or may not be measurable by contemporaneous scientific methodologies. In artificial intelligences, mental illnesses may similarly arise from conscious robots having adaptive or maladaptive responses to external exposure, or alternatively may suffer from these from their inherent human design. In both cases of humans and AI’s, the genesis of mental illness may trigger a vicious cycle that may irrevocably alter mental functioning. Although a circular argument might be imputed at this sage where: if AI robots are conscious, then they may develop mental illness, and if they have mental illness, then we can confirm that they are conscious; this would act as a fallacy. I suggest a unidirectional hierarchy of mental illness existing only when consciousness is present.

## Limitations and Strengths

This discussion may be subject to several limitations, these include that the subject of metal illness in artificial intelligences and robots may be premature and even speculative or unfalsifiable in the current era. As a result there is a conceptual vacuum where the application of philosophical matters may offer the early framework to develop an argument. As there is no consensus on the aetiology of mental disease or whether the “mental” can truly be extricated from the “physical,’ this prototype philosophical discussion to decipher whether robot behavioural disorders would be “mental” or “physical” may generate some fresh insights. The future of this field therefore is to extend our understanding of the nature and aetiology of human behavioural and mental disorders, whilst concurrently increasing our underlying perception of current and future robot and artificially intelligent mental functionaries. This may offer us the comparative insight into the mechanisms and processes of mental dysfunction. One potential strength of this discussion however is by offering urgency and noting that behavioral disorders in robots could be dangerous not just to themselves but to humanity in general, and then bring out how this may be an issue for roboethics to consider.

## Conclusions

From a philosophical point of view it can be argued that it is essentially impossible to define if anything other than oneself is conscious, however from a practical angle, the development of technologies that manifest conscious minds in the form of artificial intelligences continues to make numerous forward strides. If these artificial minds function to offer consciousness, then they also have the potential to become dysfunctional so that they may exhibit mental illness. It is important to avoid any circular fallacies such as consciousness leads to mental disease and robotic mental dysfunction therefore leads to consciousness, rather consciousness may be a state that unidirectionally can result in a subgroup of its individuals suffering from mental dysfunction; and such dysfunction cannot exist in the absence of a conscious agent. If we can acknowledge conscious AI’s, then we consequently need to acknowledge any potential mental illnesses that they develop. These would be eligible for the same rights and support that humanity dictates for those with mental illness, so that AI’s that demonstrate mental illness should be afforded the appropriate therapies that humane society can offer them. Although post hoc, the very diagnosis of mental illness in an AI may be the route of affirming the existence of conscious AIs (at least in the subgroup suffering from mental dysfunction); its existence may also offer insights into AI mind functioning in a similar manner that human mental disease can offer mechanistic insights into the human brain such that AI mental illnesses may be able to offer selected insights into some artificially intelligent minds.
